# Inferior Vena Cava Syndrome due to Giant Hepatic Cyst After Blunt Trauma

**DOI:** 10.1155/crgm/8238996

**Published:** 2026-10-09

**Authors:** Akinori Sekizawa, Yosuke Ono, Takuto Kaji, Tatsuto Shimizu, Yuta Kawahara, Kazuki Kobayashi, Takazumi Tsunenari, Hiroshi Takazaki, Takahiro Einama, Yasuhiro Obuchi, Kenichi Hashimoto, Yoji Kishi, Nobutaka Hirooka

**Affiliations:** ^1^ Department of General Medicine National Defense Medical College 3-2 Namiki Tokorozawa 359-0042 Saitama Japan ndmc.ac.jp; ^2^ Department of Surgery National Defense Medical College 3-2 Namiki Tokorozawa 359-0042 Saitama Japan ndmc.ac.jp; ^3^ Division of Clinical Training Management Medical Education and Clinical Training Center National Defense Medical College 3-2 Namiki Tokorozawa 359-0042 Saitama Japan ndmc.ac.jp

**Keywords:** blunt abdominal trauma, giant hepatic cyst, inferior vena cava syndrome, laparoscopic deroofing, lower extremity edema, simple hepatic cyst

## Abstract

A 78‐year‐old woman presented with sudden‐onset lower extremity edema, abdominal pain, and loss of appetite that developed the day after she fell off her bicycle. Computed tomography showed a simple hepatic cyst, 17 cm in diameter, compressing the inferior vena cava (IVC). Her symptoms resolved following laparoscopic deroofing. Notably, the cyst had remained stable at 12 cm 2 and 4 years prior, without any evidence of IVC compression. Although many guidelines for asymptomatic simple hepatic cysts published to date do not recommend treatment or follow‐up regardless of size, our experience suggests the importance of monitoring simple hepatic cysts larger than 12 cm.

## 1. Introduction

Simple hepatic cysts are benign lesions that are being identified more frequently, with a reported prevalence of 15%–18% [1]. Most cysts are small and asymptomatic; however, in 5% of the cases, complications such as hemorrhage, infection, or rupture may arise and occasionally necessitate emergency intervention [2]. Intracystic inflammation due to necrosis of the epithelial lining and microbleeding in the cyst wall, caused by these complications or by abdominal trauma, can result in cystic fluid production and cyst enlargement. Some enlarge to become giant cysts exceeding 10 cm in diameter. Complications from vessel compression, such as Budd–Chiari syndrome or inferior vena cava syndrome (IVCS), are severe but rare. Thus, many guidelines for asymptomatic simple hepatic cysts published to date do not recommend routine imaging follow‐up or treatment, regardless of size [3–7]. What appears to be the first documented case of IVCS caused by an enlarged giant simple hepatic cyst after blunt abdominal trauma is reported.

## 2. Case Presentation

A 78‐year‐old Japanese woman with a simple hepatic cyst was referred to our hospital for further evaluation of lower extremity edema, right upper abdominal pain, and appetite loss that had persisted for 2 months. She had no medical or family history of chronic diseases such as diabetes mellitus, connective tissue disease, or malignancy, including lymphoma. She had been taking only amlodipine 5 mg and esomeprazole 10 mg regularly, and there was no history of steroid or immunosuppressant use. On computed tomography (CT) during her medical checkup, an irregular nodule of 17 mm in size with bronchial dilation in the right upper lung lobe and a simple hepatic cyst were noted 4 years and 2 years earlier; however, they were left untreated or not further evaluated because of the lack of progression. Two months before her first visit to our hospital, she fell off her bicycle three times on the same day and hit both sides of her upper abdomen. The day after she fell off her bicycle, she developed dull right upper abdominal pain, bloating, bilateral lower extremity edema, and appetite loss. For 3–5 days after the fall, intermittent nighttime fever up to 38.7°C occurred but resolved spontaneously without treatment. Her family physician prescribed furosemide 20 mg and Saireito, a Kampo medicine used for edema; however, neither was effective.

She presented to our hospital ambulatory. At the initial visit, her temperature was 36.3°C, blood pressure was 145/70 mmHg, heart rate was 115 beats per minute, and oxygen saturation on room air was 95%. On physical examination, tenderness in the right upper abdomen, right lateral chest, and abdominal wall without guarding was noted, along with edema of the lower abdomen and lower extremities.

Laboratory test results showed mild inflammation, anemia, hypokalemia, liver dysfunction with hypoalbuminemia, coagulation dysfunction, and elevated D‐dimer levels (Table 1). Urinalysis showed no significant abnormalities.

**TABLE 1:** Laboratory findings before surgery.

	Patient result	Normal range
White blood cell count	7300/μL	3300–8600/μL
Neutro.	72.1%	38.5%–80.5%
Lymph.	18.6%	16.5%–49.5%
Mono.	7.5%	2.0%–10.0%
Eosino.	1.1%	0.0%–8.5%
Baso.	0.7%	0.0%–2.5%
Hemoglobin	10.4 g/dL	13.7–16.8 g/dL
Platelet	30.6 × 104/μL	15.8–34.8 × 104/μL
Total bilirubin	0.76 mg/dL	0.2–1.2 mg/dL
AST	24 U/L	8–30 U/L
ALT	16 U/L	5–35 U/L
Alkaline phosphatase	137 U/L	38–113 U/L
γ‐Glutamyl transpeptidase	119 U/L	7–40 U/L
Total protein	6.8 g/dL	6.5–8.2 g/dL
Serum albumin	2.3 g/dL	3.8–5.2 g/dL
Total cholesterol	123 mg/dL	130–230 mg/dL
Glucose	112 mg/dL	65–110 mg/dL
Amylase	47 U/L	29–132 U/L
BUN	7 mg/dL	8–20 mg/dL
Creatinine	0.54 mg/dL	0.61–1.13 mg/dL
Serum sodium	142 mmol/L	135–147 mmol/L
Serum potassium	2.6 mmol/L	3.5–5.0 mmol/L
Serum chloride	100 mmol/L	98–108 mmol/L
Serum iron	10 μg/dL	70–160 μg/mL
Total iron binding capacity	179 μg/dL	220–420 μg/mL
Ferritin	256.0 ng/mL	23–250 ng/mL
Brain natriuretic peptide	39.8 pg/mL	< 18.4 pg/mL
Erythrocyte sedimentation rate	76 mm/h	1–7 mm/h
C‐reactive protein	7.1 mg/dL	< 0.3 mg/dL
Carcinoembryonic antigen	4.8 ng/mL	< 5.3 ng/mL
Carbohydrate Antigen 19–9	89.6 U/mL	< 37 U/mL
PT‐INR	1.17	0.9–1.1
aPTT	33.8 s	24.0–32.0 s
D‐dimer	5.0 μg/mL	< 1.0 μg/mL

However, chest radiography showed elevation of the right hemidiaphragm, pleural effusion, obliteration of the right heart border, and leftward displacement of the cardiac silhouette (Figure S1). Contrast‐enhanced CT demonstrated a smooth, well‐circumscribed, oval cyst with a thin wall, measuring 169 × 140 × 165 mm^3^, that compressed the inferior vena cava (IVC), along with slight dilation of the intrahepatic bile ducts, diffuse mesenteric edema, and ascites (Figure S2a,b). No elliptical area of hypodensity was seen around the cyst. Retrospective review of CT images during her medical checkup showed that the cyst had grown rapidly from 118 × 95 × 109 and 123 × 96 × 113 to 169 × 140 × 165 mm^3^ over the last 2 years. The CT number of the cyst fluid on plain CT also increased, from −0.9 and 0.5 to 12.1 (Figure S2c–f). Given the presence of edema, liver dysfunction, hypokalemia, elevated D‐dimer levels, and the CT findings of ascites and IVC enlargement, the tentative diagnosis was IVCS due to an enlarged hepatic cyst. Hepatic hematoma, biloma, infectious or hemorrhagic simple hepatic cysts, type 1 hydatid cyst, and a hepatic cystic neoplasm were included in the differential diagnosis of a cyst that had enlarged rapidly. Because she was stable and able to perform activities of daily living independently, she was evaluated for malignancy and provided with symptomatic treatment with careful outpatient follow‐up.

Magnetic resonance imaging (MRI) showed a homogeneous cyst with high signal intensity on T2‐weighted images, along with a nonenhancing nodular area in the dorsal region that showed low signal intensity on T2‐weighted images and high signal intensity on diffusion‐weighted images, findings suggestive of intracystic hemorrhage or clot formation. Absence of part of the cyst wall and protrusion of the cyst suggesting necrosis of the cyst wall and rupture of the cyst were also evident (Figure S3a–c). No hypointense peripheral ring on T2‐weighted images or features suggestive of malignancy were identified. Magnetic resonance cholangiopancreatography (MRCP) showed no communication between the cyst and the biliary ductal system (Figure S3d).

Regarding the treatment of symptomatic simple hepatic cysts, no high‐quality studies or guidelines recommending a particular approach have been reported to date. Percutaneous aspiration and sclerotherapy may be less invasive; however, they are associated with higher recurrence rates and more treatment‐related adverse events when sclerosing agents are used. Because the liver surrounding the cyst was thin, treatment with laparoscopic deroofing rather than percutaneous aspiration was selected in the present case.

Two weeks after the initial visit, laparoscopic deroofing of the cyst using indocyanine green fluorescence navigation was performed. Intraoperatively, pus‐like fluid was aspirated (Figure 1), and culture identified *Klebsiella pneumoniae*. Histopathological examination of the cyst wall showed sloughing of the epithelial lining, dense fibrous tissue, and marked inflammatory cell infiltration, without evidence of malignancy or Echinococcus organisms, confirming the diagnosis of a nonparasitic, simple hepatic cyst with acute inflammation.

**FIGURE 1:**
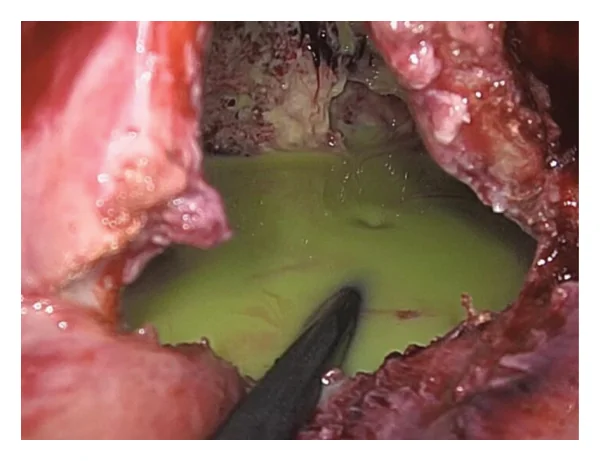
Intraoperative image demonstrating a cyst containing pus‐like fluid.

The patients’ postoperative course was uneventful, without any bile leakage, and she was discharged on postoperative Day 5. During postoperative follow‐up, the lower extremity edema resolved gradually, and the patient’s appetite returned. No recurrence of the hepatic cyst was found during 10‐month follow‐up.

## 3. Discussion

Asymptomatic simple hepatic cysts are typically managed with a watchful waiting approach, with no need for routine surveillance or intervention [3–7]. However, complications such as cyst enlargement, infection, or rupture may present with acute right upper quadrant and referred shoulder pain [3, 4]. In the present case, symptoms, including pain, fever, and edema, developed the day after abdominal trauma. Simple hepatic cysts are believed to be congenital [8] and typically remain stable or decrease in size over several years [9]. However, the cyst in the present case, which had been stable 4 years and 2 years earlier, tripled its volume over the 2 years prior to her presentation. Therefore, it was considered that the abdominal trauma 2 months before her first visit caused or accelerated enlargement of the cyst. In a case of traumatic rupture of a nonparasitic simple hepatic cyst reported by Jinyoung, epigastric pain occurred immediately after trauma [10]. Koc et al. reported rupture of hydatid cyst caused by severe inflammation with elevation of the white blood cell (WBC) count over 10,000/mm^3^ and an allergic reaction [11]. Absence of part of the cyst wall and protrusion of the cyst on MRI suggested rupture; nevertheless, her onset was delayed, and her WBC count was within normal limits. This suggested that the blunt force did not directly cause rupture but rather caused inflammation, increased intracystic pressure, and injury to fragile capillaries within the cyst wall, leading to necrosis of the epithelial lining and collapse of the cyst wall [12]. The CT number of the cyst fluid increased, and the cyst fluid intraoperatively appeared to contain some bile. This finding suggested that the blunt force also caused rupture of bile ducts surrounding the cyst and the cyst wall, leading to bile flow into the cyst and enlargement of the cyst.

Characteristic imaging findings of infected hepatic cysts include fluid‐debris levels, occasional intralesional gas, peripheral rim enhancement in a thickened cyst wall, and perilesional edema or hyperemia on CT. On MRI, infected cysts may appear hyperintense on T1‐weighted images, with surrounding hyperintense perilesional edema on T2‐weighted images [13]. However, the cyst in the present case exhibited homogeneity, with a thin, nonenhancing wall, and lacked perilesional edema, despite *Klebsiella* infection. The possible entry routes for infection were biliary entry via cystobiliary communication, portal entry including bacterial translocation, and hematogenous transmission. Because laboratory, urinalysis, and CT findings suggested no site of infection such as an abscess, peritonitis, nephritis, or cholecystitis, hematogenous transmission was considered unlikely. Mizumoto et al. reported that cystobiliary communication was found in only 2 of 10 cases of infected hepatic cysts associated with cholangitis or biliary interventions [14]. CT findings, MRCP findings, intraoperative findings, and the bilirubin level of the peritoneal fluid from the drainage tube suggested no continuous bile leakage; however, the existence of a small cystobiliary communication or the possibility of closure of the communication due to inflammation from cyst infection was probable. Endoscopic retrograde cholangiography should have been considered. We hypothesized that the infection occurred immediately before surgery due to bacterial translocation associated with persistent intestinal and colonic edema, and immunosuppression related to malnutrition and liver dysfunction may have increased the patient’s susceptibility to infection.

IVCS caused by simple hepatic cysts has been reported on multiple occasions, with 10 cases documented to the best of our knowledge [15–24] (Table 2). Notably, all reported cysts were located in the right hepatic lobe, with most exceeding 13 (range, 7–20) cm in diameter on CT. In the case reported by Panchal et al. [18], the cyst causing IVCS, measuring 89 × 96 mm^2^ 10 years prior, did not show contact with the IVC. In the present case, CT performed 2 and 4 years before the development of IVCS showed narrowing of the IVC. However, the family doctor who ordered the CT during her medical checkup, the radiologist who reviewed the images, and the primary care physician who referred her to our hospital did not notice that her IVC was narrowed or compressed. One of the reasons for overlooking this finding may be the preconception that simple hepatic cysts are benign and require no treatment or careful follow‐up for enlargement. Therefore, though management of asymptomatic simple hepatic cysts typically does not require follow‐up or treatment, we propose that close monitoring or decompression for cysts over 12 cm in diameter should be considered to prevent the development of IVCS.

**TABLE 2:** Ten reported cases of IVCS due to simple hepatic cysts.

Reference	Age/sex	Major complaint	Cyst location	Size of cyst	Treatment	Outcome
Park [10]	79/F	Foot and ankle edema	Right lobe	19 × 14 cm	Laparoscopic fenestration and drainage	5 days, discharged
Koc et al. [11]	80/M	Lower extremity edema and dyspnea	Right lobe	176 × 190 mm	Laparoscopic deroofing and drainage	2 months, no symptoms
Shimizu et al. [12]	88/F	Right abdominal pain and lower extremity edema	Right lobe	17 cm	Percutaneous drainage and sclerotherapy	1 week, relieved
Morii et al. [13]	68/F	Right upper quadrant pain	Right lobe	14 × 15 × 18 cm	Laparoscopic deroofing	Not described
Mizumoto et al.[14]	84/F	Weight loss, loss of appetite, abdominal distension, and lower extremity edema	Right lobe	19 cm	Laparoscopic deroofing	No symptoms
Ji et al. [15]	69/F	Right lower extremity edema	Segment 1	6 × 10 × 7 cm	Percutaneous drainage	6 month, relieved
Nakabayashi et al. [16]	67/M	Shortness of breath	Right lobe	20 cm	Laparoscopic resection	Not described
Ko et al. [17]	83/F	Fatigue and loss of appetite	Right lobe	Not described	Laparoscopic deroofing	2 months, no symptoms
Panchal et al. [18]	70/F	Right lower extremity edema and pain	Right lobe	17 cm	Percutaneous drainage	3 weeks, relieved
Nicolau et al. [19]	56/F	Dyspnea	Right lobe	17 × 13 cm	Surgical evacuation	1 year, no symptoms

In conclusion, the first case of IVCS caused by an enlarged giant simple hepatic cyst following blunt abdominal trauma was described. IVCS caused by hepatic cysts is rare but potentially severe; therefore, clinicians, particularly in primary care, should consider IVCS in patients with giant hepatic cysts who present with lower extremity edema, especially after abdominal trauma.

## Author Contributions

Akinori Sekizawa: conceptualization, data curation, project administration, writing–original draft, and writing–review and editing. Yosuke Ono: conceptualization, supervision, and writing–review and editing. Takuto Kaji: supervision and writing–review and editing. Tatsuto Shimizu: supervision and writing–review and editing. Yuta Kawahara: supervision and writing–review and editing. Kazuki Kobayashi: supervision and writing–review and editing. Takazumi Tsunenari: supervision and writing–review and editing. Hiroshi Takazaki: supervision and writing–review and editing. Takahiro Einama: supervision and writing–review and editing. Yasuhiro Obuchi: supervision and writing–review and editing. Kenichi Hashimoto: supervision and writing–review and editing. Yoji Kishi: supervision and writing–review and editing. Nobutaka Hirooka: conceptualization, supervision, and writing–review and editing. All authors were involved in clinical management of the patient.

## Funding

The authors received no specific funding for this work.

## Disclosure

An earlier version of this manuscript was published as a preprint on *Authorea* (doi: 10.22541/au.175404816.68787512/v1) [25].

## Ethics Statement

This study was reviewed and approved by the Ethics Committee at National Defense Medical College in Japan (No. 5401) and was conducted in compliance with the Declaration of Helsinki (https://www.wma.net/policies-post/wma-declaration-of-helsinki/). The patient provided written, informed consent to participate in this study. Written, informed consent was also obtained from the patient for the publication of any potentially identifiable images or data included in this article.

## Conflicts of Interest

The authors declare no conflicts of interest.

## Supporting Information

Additional supporting information can be found online in the Supporting Information section.

## Supporting information


**Supporting Information** Figure S1. Chest radiograph showing elevation of the right diaphragm and pleural effusion. Figure S2. Computed tomography at the first visit showing an oval cyst measuring 169 × 140 × 165 mm^3^ compressing the inferior vena cava (IVC) (a) and diffuse mesenteric edema and ascites (b). The cyst measuring 118 × 95 × 109 mm^3^ 4 years earlier (c, d) and 123 × 96 × 113 mm^3^ 2 years earlier (e, f). The IVC is constricted (white arrows). The CT numbers of cyst fluid are −0.9 4 years earlier, 0.5 2 years earlier, and 12.1 at the first visit. Figure S3. Magnetic resonance imaging showing a homogeneous cyst with high signal intensity on T2‐weighted images (a) and diffusion‐weighted images (b). A nodular region suggesting intracystic hemorrhage or clot formation is noted (black arrows). Absence of part of the cyst wall and protrusion of the cyst are also present (c). Magnetic resonance cholangiopancreatography showing no direct communication between the cyst and the biliary duct system (d).

## Data Availability

The data that support the findings of this study are available from the corresponding author upon reasonable request.
